# Hybrid exciton-plasmon-polaritons in van der Waals semiconductor gratings

**DOI:** 10.1038/s41467-020-17313-2

**Published:** 2020-07-15

**Authors:** Huiqin Zhang, Bhaskar Abhiraman, Qing Zhang, Jinshui Miao, Kiyoung Jo, Stefano Roccasecca, Mark W. Knight, Artur R. Davoyan, Deep Jariwala

**Affiliations:** 1 Department of Electrical and Systems Engineering, University of Pennsylvania, Philadelphia, PA 19104 USA; 2 Department of Physics, University of Pennsylvania, Philadelphia, PA 19104 USA; 3 Department of Electrical and Computer Engineering, National University of Singapore, Singapore, 117583 Singapore; 4 NG Next, Northrop Grumman Corporation, Redondo Beach, CA 90278 USA; 5 Department of Mechanical and Aerospace Engineering, University of California, Los Angeles, CA 90095 USA

**Keywords:** Engineering, Materials science, Nanoscience and technology, Optics and photonics, Physics

## Abstract

Van der Waals materials and heterostructures that manifest strongly bound exciton states at room temperature also exhibit emergent physical phenomena and are of great promise for optoelectronic applications. Here, we demonstrate that nanostructured, multilayer transition metal dichalcogenides (TMDCs) by themselves provide an ideal platform for excitation and control of excitonic modes, paving the way to exciton-photonics. Hence, we show that by patterning the TMDCs into nanoresonators, strong dispersion and avoided crossing of exciton, cavity photons and plasmon polaritons with effective separation energy exceeding 410 meV can be controlled with great precision. We further observe that inherently strong TMDC exciton absorption resonances may be completely suppressed due to excitation of hybrid light-matter states and their interference. Our work paves the way to the next generation of integrated exciton optoelectronic nano-devices and applications in light generation, computing, and sensing.

## Abstract (web summary)

The authors investigate the optical properties of a heterostructure formed by a metallic substrate and a nanostructured transition metal dichalcogenide multilayer by measuring the reflectance spectrum at different multilayer thicknesses, filling factors and grating periods. The spectra show strong dispersion and avoided crossing of excitons, plasmons and cavity photons along with excitonic mode suppression at the anti-crossing point.

## Introduction

**Fig. 1:**
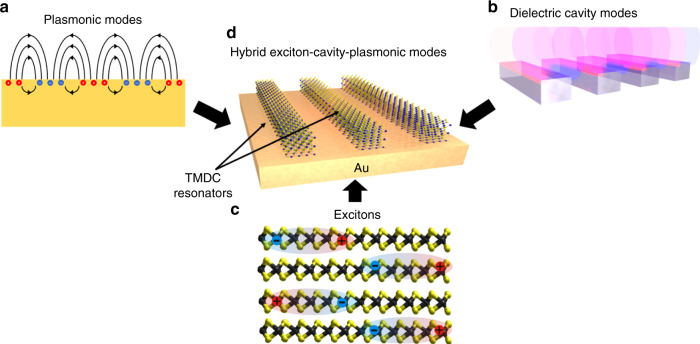
Conceptual illustration of multipartite light–materials interaction within nanoscale materials and patterned structures. Light interaction with nanostructured metals (**a**) and dielectrics (**b**) gives rise to the excitation of plasmonic and dielectric-cavity resonances, respectively. Dispersion of these resonances is determined mainly by the geometry of the system. **c** Reduced dimensional materials provide a different approach to strong light–materials interaction. Here, sharp optical resonances manifest in the excitation of long-lived bound electron-hole pairs, with layered two-dimensional transition metal dichalcogenides (TMDCs) as prominent examples. **d** By mixing engineered geometric dispersion in metal-dielectric nanostructures with intrinsic excitonic resonances in TMDCs, a multifaceted, strongly-interacting interplay of photonic and electronic states can be achieved.

Control of light-wave dispersion at the nanoscale is of great importance for applications ranging from lasing to sensing to computing. Plasmonic and high contrast dielectric nanostructures^1–4^ are a frontier approach for dispersion engineering (Fig. 1a, b) at the dimensions comparable to and smaller than the light wavelength. Materials with resonant quantum confined states provide an alternative route for controlling light propagation and interaction. This strategy has been effectively employed in a wide variety of materials ranging from inorganic III–V epitaxial quantum wells to organic small molecules and polymers, to even carbon nanotubes, and most recently, two-dimensional (2D) transition metal dichalcogenides (TMDCs) (Fig. 1c). Due to weak intermolecular bonding or physical confinement, excitons in this class of materials exhibit strong binding energies and dominate optical responses even at room temperatures^5^. Among these excitonic materials, the 2D TMDCs of Mo and W are of particular interest since the strong exciton binding manifests in high refractive indices and extinction coefficients (k) near the exciton resonance. Light–exciton interaction has been extensively studied in monolayer direct-band gap TMDC films^6–11^. These studies have involved coupling the monolayers to extrinsic plasmonic or dielectric optical meta-elements or cavities or metasurfaces^12–14^. Strong coupling regimes and families of hybrid exciton modes have been observed. Nevertheless, light-interaction with monolayer films is challenged by the very large disparity between optical wavelength and film thickness. Recent demonstration of enhanced resonant light interaction with few-layer thick TMDCs suggests that thin systems may be well suited for light dispersion^5,15–17^.

Here, we show that nanostructured multilayer TMDCs provide a natural platform for taming light–exciton interaction. Specifically, within a single integrated platform we observe multipartite interaction and hybridization, leading to strong coupling between three resonant excitations with an avoided crossing of 410 meV at room temperature. We further reveal a regime of light-TMDC interaction associated with suppression of inherent exciton absorption resonances due to interference of hybridized states at the nanoscale.

## Results

### Nanopatterned multi-layer WS_2_ grating resonators on Au

**Fig. 2:**
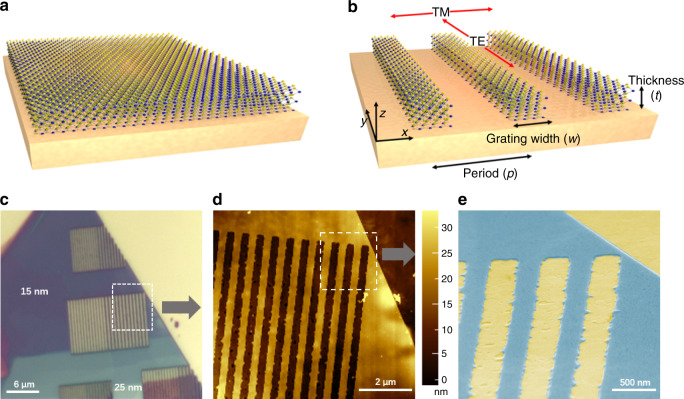
Nanopatterned multi-layer WS_2_ grating resonators on gold. **a** Schematic of an unpatterned multi-layer WS_2_ flake on an Au substrate. **b** Schematic of a multi-layer WS_2_ grating structure on a gold substrate. Red arrows denote the incident light polarization: TM polarization is defined such that the electric field is perpendicular to the grating, while the TE polarization electric field is parallel to the grating. Grate width (*w*), period (*p*), and thickness (*t*) are defined. **c** Unpolarized white light micrograph of WS_2_ flake on Au substrate, showing patterned and unpatterned regions. Different colors appear due to different absorption spectra for different material thicknesses; 15 nm thick WS_2_ exhibits a purple hue while 25 nm thick WS_2_ appears pale blue. **d** Atomic force microscopy (AFM) image of the grating topography corresponding to dashed box of (**c**). **e** False color scanning electron microscopy (SEM) image of the boxed region in panel d acquired at 70° tilted angle. The grating structure with dimensions *w* = 300 nm and *p* = 600 nm is clearly seen.

We begin by systematically exploring optical reflectance spectroscopy, first in unpatterned (Fig. 2a) and then in patterned cases (Fig. 2b) of varying TMDC thickness. Our choice of the TMDC is WS_2_, but our results are generalizable to other room temperature excitonic materials (see Supplementary Note 1). Micromechanically exfoliated WS_2_ flakes were obtained on a template-stripped Au substrate by mechanical exfoliation from bulk crystals using a tape^18^. The flakes were directly transferred using the tape onto the template stripped Au substrates. Additional details of sample preparation are provided in the “Methods” section. Upon exfoliation, these flakes exhibit thickness-dependent colors resulting from Fabry–Perot-like resonances^16^, resulting in translucent red, purple, blue, and white regions of crystal (Fig. 2c). Periodic one-dimensional (1D) gratings were etched in single WS_2_ flakes to increase the light–materials interaction strength and engineer optical dispersion. Additional fabrication details and measurement methods are provided in Supplementary Note 2. Structural characterization of grating etch patterns by atomic force microscopy (AFM) and scanning electron microscopy (SEM) (Fig. 2c, d) shows deep-subwavelength WS_2_ flake thicknesses, along with gratings etched through the full flake. The etching process results in some edge roughness, but these non-perturbative structural deviations do not modify the optically critical parameter, which is the grating period. Detailed optical micrographs, AFM profiles and SEM images are shown in Supplementary Note 2.

### Resonant absorption in nanopatterned WS_2_ on Au

**Fig. 3:**
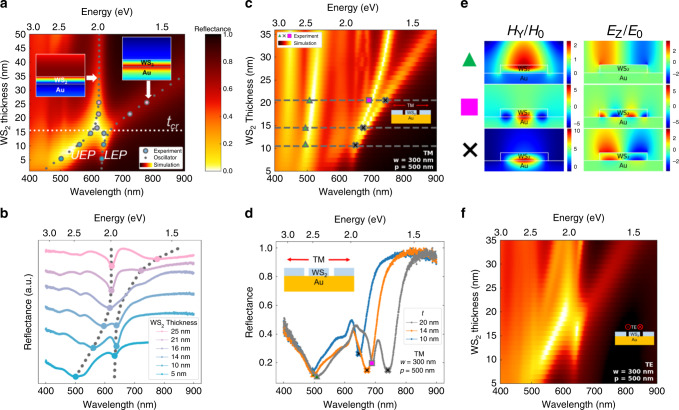
Below-gap resonant absorption in WS_2_ on Au. **a** Plot of simulated reflectance spectra of unpatterned WS_2_ on Au with varying WS_2_ thickness on the *y* axis. The primary exciton mode of WS_2_ can be seen at ~2.0 eV which couples with the cavity mode, splitting the exciton into the upper exciton-polariton (UEP) and the lower exciton-polariton (LEP). A low Q Fabry–Perot-like cavity is formed even in deep-subwavelength thickness regime as a result of the thin-film interference. Insets show the *H*_Y_ field profiles corresponding to the exciton mode (left) and the cavity mode (right). Reflectance color scale is the same for (**a**, **c**, **f**). Dashed lines represent a model of two coupled oscillators, while circles represent experimentally measured peak positions shown in (**b**). **b** Experimental unpolarized reflectance spectra of unpatterned WS_2_ on Au with varying thickness. The experimentally measured thicknesses are 5, 10, 14, 16, 21, and 25 nm. The UEP and LEP peaks are emphasized with circles and are superimposed on panel a to show strong matching with simulation. **c** Simulated reflectance spectra under TM polarization of a 1D WS_2_ grating on an Au substrate with varying thickness. The structure has a fixed width and period of 300 nm and 500 nm, respectively. The non-dispersive mode induced by dielectric grating emerges as the highest-energy resonance (~500 nm) while plasmon resonances of varying orders emerge at lower energies. For higher thickness regimes, an additional plasmon resonance emerges which red-shifts with increasing thickness. **d** Experimental TM reflectance spectra of the 1D WS_2_ grating on the Au substrate with *w* = 300 nm and *p* = 500 nm. The peaks corresponding to various modes are coded with matching symbols on (**c**). **e** *E*_Z_ and *H*_Y_ mode profiles for the various modes matching with the corresponding symbols in (**c**, **d**). **f** Simulated reflectance spectra under TE polarization of a 1D WS_2_ grating on an Au substrate as thickness is swept.

Reflectance spectra for unpatterned WS_2_ of varying thickness on an Au back reflector (Fig. 3a, b) exhibit two reflectance dips (absorption resonance peaks). The primary exciton mode of WS_2_ at 2.0 eV demonstrates high absorption independent of sample thickness and is therefore the focus of this study. While excitons which possess out-of-plane dipoles have also been predicted and observed in monolayer TMDCs^19^ that have a direct band-gap, the multilayer samples in this study combined with normal incidence illumination prevents any in-coupling of light in reflectance measurements or out-coupling of light in photoluminescence measurements (see Supplementary Note 3). The simulated magnetic field profile indicates strong exciton driven light absorption, suggesting Beer–Lambert like absorbance, typical for bulk samples. Additional details on simulation methods are provided in the “Methods” section. In contrast, the magnetic field profile representing the Fabry–Perot resonance indicates a low-Q cavity mode formed by the combination of metal and high-index dielectric in the heterostructure^16,20^. The cavity mode is observed for deep-subwavelength thicknesses, owing to the lossy dielectric of WS_2_ on a reflective substrate facilitating near-unity absorptive resonances. The cavity mode couples to the primary exciton mode at the critical thickness ~15 nm, resulting in overall absorption enhancement. This coupling results in hybridization of the cavity photon and exciton modes into exciton-polariton modes with a characteristic avoided crossing in the reflectance spectra. As the cavity mode is tuned through the exciton energy, the upper exciton-polariton (UEP) branch and lower exciton-polariton branch (LEP) are split^21^. At the point of avoided crossing of the exciton and the cavity photon, the splitting energy is evaluated to be *ħΩ* = 170 meV by fitting the simulation data to a coupled oscillator model (Supplementary Note 4). Experimental reflectance spectra with varying thicknesses of WS_2_ show remarkably good qualitative and quantitative agreement with the simulations (Fig. 3b). Both the primary exciton peak and the weak B exciton peak (*λ* ~ 515 nm) locations match well. Note that this coupling between a weak cavity mode and an exciton mode in the deep subwavelength thickness regime can be extended to other TMDC materials and geometries. The coupling also extends to higher order cavity modes for larger thicknesses of WS_2_ (see Supplementary Note 5). Notably, such behavior cannot be observed in III–V semiconductors (e.g., GaP or GaAs) that possess similar band gaps but lack room-temperature excitonic features in their dielectric functions (see Supplementary Note 1). We further note that the optical constants used in our simulations are assumed constant as a function of thickness and are derived from bulk optical constants of WS_2_^5^. This is because the phenomena reported in this study are focused in the range of 8–25 nm thickness which represents the electronic bulk in WS_2_^22^ as the effects of electronic quantum confinement become negligible at ~5 nm (6–7 layer) thickness.

Periodic patterning provides an additional degree of control for light–exciton interaction and dispersion engineering (Fig. 3c, d). Under TM polarization, we observe that several additional absorption resonances emerge for a periodically patterned TMDC. Primarily, a resonant mode associated with a dielectric grating emerges at the highest-energies (~500 nm) while several plasmonic modes are excited at lower energies due to the change in sign of the real permittivity across the WS_2_/Au interface (Fig. 3e)^23,24^. Characteristic mode profiles are shown in Fig. 3e. Different types of plasmonic mode localization and confinement are observed. For higher thicknesses (more than ~15 nm thick), these plasmonic modes further red-shift with increasing thickness. Detailed discussion on **E** and **H** field profiles of the modes is provided below. Our simulation results closely match experimental reflectance spectra for three discrete thicknesses of 10, 14, and 20 nm (Fig. 3d). Under TE polarization (Fig. 3f), the simulated reflectance spectra behave similarly to the unpatterned case, implying that grating and plasmon modes are not excited, since there is no breaking of symmetry along TE polarization upon etching. Experimentally measured reflectance spectra also match the simulations for the TE case, and the spectra, as well as mode profiles, are shown in detail in Supplementary Note 6. All simulations and experimental measurements are for TM polarization for the remainder of this work. We would like to note that the observed modes in this case are non-propagating unlike the prior predicted^25^ and observed^26^ guided modes in ultrathin TMDCs. This is also evident from simulations of our field profiles that do not show evidence of any guided-mode resonances.

### Plasmonic modes above critical-thickness of WS_2_

**Fig. 4:**
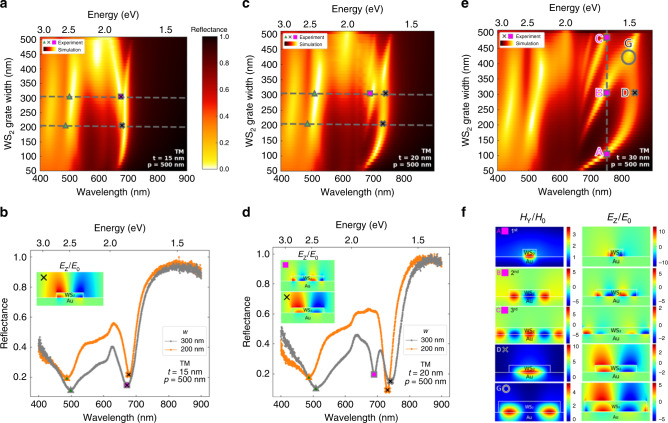
Plasmonic modes above critical-thickness of WS_2_ on Au. **a** Width dependence of simulated TM reflectance spectra. Period is fixed at 500 nm, and thickness is fixed at 15 nm (critical thickness). The plasmonic peak emerges from the lower exciton-polariton (LEP) of the unpatterned case indicating a hybrid nature of the mode. Reflectance color scale is the same for (**a**, **c**, **e**). **b** Experimental reflectance spectra of the WS_2_ grating structure with *t* = 15 nm and *p* = 500 nm, and widths of 200 nm and 300 nm. Corresponding mode peaks are coded with matching symbols in (**a**). Inset is the *E*_Z_ field profile of the first order plasmon mode. The symbols on peaks measured in experimental spectra are marked on the simulated plots in (**a**). **c** Width dependence of simulated TM reflectance spectra. Period is fixed at 500 nm, and thickness is fixed at 20 nm (above critical thickness i.e. uncoupled regime). Since the thickness is sufficient to support cavity modes in the unpatterned case, plasmon modes (marked with square symbols) start emerging from the cavity mode. **d** Experimental reflectance spectra of the WS_2_ grating structure with thickness and period fixed at 20 nm and 500 nm respectively. Spectra for widths of 200 nm and 300 nm are compared. Inset are the *E*_*z*_ field profiles of the two plasmonic modes. The symbols on peaks measured in experimental spectra are marked on the simulated plots in (**c**). **e** Width dependence of simulated TM reflectance spectra. Period is fixed at 500 nm, and thickness is fixed at 30 nm, sufficiently above the critical thickness regime. For a photon wavelength of 750 nm, the resonant widths are equal to 100 nm (A), 300 nm (B), and 470 nm (C), forming 1st, 2nd, and 3rd order plasmon modes. The plasmon mode (D) stands at the lowest-energy resonance. The mode G marked at the crossing point of the 2nd order plasmon mode (B) and (D) indicates a hybrid mode with characters of both. **f** *E*_Z_, *H*_Y_ field profiles of the plasmonic modes labeled with corresponding points in (**e**).

To understand further how the lateral patterning affects mode dispersion, we have investigated the dependence of reflection spectra on grating finger width. At the critical thickness (~15 nm), a plasmon-mode branch emerges from the LEP branch which suggests it has a hybrid character (Fig. 4a). The experimental reflectance spectra for fixed *t* = 15 nm and *p* = 500 nm, and widths of 200 nm and 300 nm match well with the simulation. This along with the non-dispersive grating induced mode and a shallow exciton-polariton mode around 600 nm are all observable in the experimental spectra (Fig. 4b). Beyond the critical thickness regime, more (higher order) plasmon modes emerge from the hybrid LEP mode (Fig. 4c) which is once again verified in the experimental spectra, (Fig. 4d) where a second peak emerges at 695 nm besides the first peak at 740 nm. Grating structures also offer a possibility of tuning frequency of these excited plasmons by changing grating geometrical parameters i.e. width. (Fig. 4e). The grating with its own wave-vector presents momentum matching conditions to excite plasmons in integer multiples of this wavevector. Higher-order surface plasmons (A,B,C in Fig. 4e) emerge in the reflectance spectra as the width is increased, while the plasmon mode indicated by D (Fig. 4d) retains its place as the lowest energy branch. The *E*_z_ and *H*_y_ field profiles show plasmons of different orders trapped between the WS_2_/Au interface (Fig. 4f) verifying our assumption. Another interesting observation that we make in our system is the occurrence of plasmonic modes emerging from the interaction of two parent modes^27,28^. At the crossing point of the plasmon mode labeled D in Fig. 4e with one labeled B, there is a minor dip in intensity followed by re-emergence of the linear dispersion, which is slightly offset from the original plasmon line. We designate these as hybrid mode G retaining character of both the B and D plasmon branches. This can be clearly verified by the mode profile in Fig. 4f where both the *H*_y_ and *E*_z_ field profiles of mode G are a combination of the field profiles of modes B and D. Extensive width dependence of these modes is shown in detail as a set of figures in Supplementary Note 7 and Supplementary video/animation 1. We observe two different types of plasmon polaritons in our system. Mode D is representative of localized resonance which is invariant of grating width whereas A, B and C have linear dependence on resonator width and hence can be referred to as surface plasmon modes. This suggests that modes A, B and C are excited by the dielectric discontinuity created by the WS_2_ (ε > 0) resonator at the Au (ε < 0)/air surface and are confined at the interface between Au and WS_2_. Hence their resonances occur at widths equal to odd integers of half of resonance wavelengths as also evident from their field profiles in Fig. 4f. Note that the plasmons observed in our system may be correlated to different types of plasmon modes (e.g., localized and surface plasmons)^1^. However, here due to the complex nature and multipartite interactions involved, we avoid making any such categorical classifications.

### Strong three-oscillator coupling below critical thickness

**Fig. 5:**
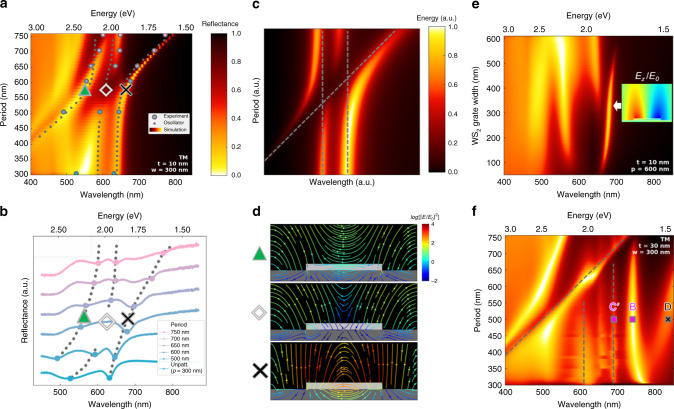
Strong three-oscillator coupling below critical thickness. **a** Period dependence of simulated TM reflectance spectra. Thickness and width are fixed at 10 nm and 300 nm, respectively. Period is swept from the unpatterned case (*w* = *p* = 300 nm) to a period of 750 nm. The dashed lines represent the calculated absorption spectra based on the three-oscillator model in (**c**), while the circles represent experimentally measured peak positions from (**b**). **b** Experimental reflectance spectra of the WS_2_ grating structure with a fixed thickness of 10 nm and a width of 300 nm. Spectra for the patterned case, *p* = 500 nm, 600 nm, 650 nm, 700 nm, and 750 nm are plotted and offset for clarity. The marked peaks are also plotted in (**a**), for comparison with simulation. **c** The calculated energy dispersion relation based on the three-oscillator model. The three oscillators represent the grating mode, LEP mode, and UEP mode. The bare modes are represented by dashed lines in the figure. By fitting the oscillator model to the simulation and experiment data, we obtain an effective mode separation energy of 410 meV. **d** Comparison of the field-line profiles of the UP, MP cut-off, and LP regions marked with triangles, diamonds and crosses, respectively in (**a**, **b**). At the point marked with a diamond, almost all the electric field lines are repelled out of the resonator rendering the highly lossy WS_2_ resonator invisible in the far-field. **e** Simulated reflectance spectra of grating resonators showing the evolution of the strong-coupling-induced separation as a function of width in the wavelength range of ~550–650 nm. Inset: the *E*_Z_ field profile of the plasmonic mode for *t* = 10 nm. **f** Period dependence of reflectance of the WS_2_ grating structure for *t* = 30 nm and *w* = 300 nm. The three dashed lines representing the bare modes are also shown in the figure, indicating the exciton mode at *λ* = 610 nm almost vanishes. The dielectric grating induced mode is unable to couple with the pure exciton without the exciton being hybridized with the cavity photon.

The above discussion summarizes the emergence of various plasmonic modes above critical thickness and their hybridization with each other. Thus far there has been no mention of the dielectric grating mode and its interaction with the exciton. This interaction is strong and notably observed for thicknesses below the critical value. The incident wave couples to the guided mode, and the dielectric-grating mode is formed under the momentum matching condition between the incident light-frequency (*ω*) and in-plane wave momentum (*k*_x_) component generated by the 1D dielectric (WS_2_) gratings^24,26^. The dielectric grating induced mode couples the incident light in-plane which allows us to estimate a momentum vs frequency dispersion of the resonances. Below the critical thickness (*t* = 15 nm, or in the strong-coupling regime), the original primary exciton absorption peak is split into LEP (~630 nm) and UEP (~550 nm) modes as a result of strong coupling between the exciton and the cavity mode, as shown in Fig. 5a for the case of 10 nm thick WS_2_. Both the UEP and the LEP can further couple to the dielectric grating induced mode, forming a three-oscillator coupling^29^. The three-oscillator plasmon-exciton polaritons coupling has also been reported in several previous works^30–32^. In a structure with a fixed width of 300 nm and thickness of 10 nm WS_2_, the grating mode can be tuned by varying the period to be in resonance with both UEP and LEP modes. It is worth noting that the UEP and LEP, while coupled as seen in the Fig. 3a, b of the unpatterned case, are period independent unless they interact with the linearly dispersive grating mode. Upon the interaction of grating modes with the two coupled branches of polaritons, strong-coupling occurs among all three modes, leading to redshift of both UEP and LEP modes. The grating mode merges into the non-dispersive upper polariton branch (UPB), while the lower polariton branch (LPB) switches from the non-dispersive LEP mode to plasmonic mode with increasing period. The UEP mode forms the middle polariton branch (MPB), which has a cut-off in the period range of 500 nm to 650 nm. A three-oscillator model is applied to fit the system governed by Eq. (1) below. (Fig. 5c). In this equation, *E*_d_, *E*_UEP_ and *E*_LEP_ represent the resonant energies of the dielectric-grating mode, the split upper-exciton-polariton branch and the lower-exciton-polariton branch respectively. *g* represents the coupling strength between two branches (e.g., *g*_d-UEP_ represents the coupling strength between the dielectric-grating branch and the UEP branch).

1 $$\left( {\begin{array}{*{20}{c}} {E_{\mathrm{d}}} & {g_{{\mathrm{d}} - {\mathrm{UEP}}}} & 0 \\ {g_{{\mathrm{d}} - {\mathrm{UEP}}}} & {E_{{\mathrm{UEP}}}} & {g_{{\mathrm{LEP}} - {\mathrm{UEP}}}} \\ 0 & {g_{{\mathrm{LEP}} - {\mathrm{UEP}}}} & {E_{{\mathrm{LEP}}}} \end{array}} \right)\left( {\begin{array}{*{20}{c}} {\alpha _1} \\ {\alpha _2} \\ {\alpha _3} \end{array}} \right) = E\left( {\begin{array}{*{20}{c}} {\alpha _1} \\ {\alpha _2} \\ {\alpha _3} \end{array}} \right)$$

(See Supplementary Note 8 for in-depth discussion of the model and parameters). We would like to note that a similar fitting-based phenomenological description of complex multi-particle interactions has been conducted in many other experimental nanophotonic systems^33,34^. Because polaritonic coupling dominates the response of this system, we assume *g*_d−LEP_ to be zero so the effective separation energy can be expressed in terms of the coupling strengths *g*_d−UEP_ and *g*_LEP−UEP_. From the experimental spectrum corresponding to the zero-detuning point (*p* = 600 nm), *g*_d−UEP_ and *g*_LEP−UEP_ are taken to be 116 meV and 89 meV, respectively (Fig. 5b). When the dielectric grating induced mode and the plasmonic mode are closest in energy, and the MPB is cut-off in between them, the giant effective separation energy which is the maximum experimentally observed separation is estimated to be $\hbar \Omega = 2\left( {g_{{\mathrm{d}} - {\mathrm{UEP}}} + g_{{\mathrm{LEP}} - {\mathrm{UEP}}}} \right) = 410\,{\rm{meV}}$. It is worth noting that for these nanopatterned grating samples, the linewidth of the dielectric grating mode and the exciton-cavity hybrid mode are *γ* = 133 meV and *κ* = 50 meV, respectively. In comparison, the effective mode separation is 2*g* = 410 meV which is much larger than (*γ* + *κ*)/2, further supporting our evidence for strong-coupling^35^. Interestingly, the MPB cut-off is only replicated in the oscillator model when it is assumed that the MPB oscillator, i.e. the UEP mode, is the only one driven. This suggests the UEP mode is the primary excitation mechanism of this system. This cut-off of the MPB upon strong interaction between these three modes has not been observed in earlier reports of strong light–matter coupling of quasiparticles in TMDCs^36^. From the electromagnetic simulation, we observe that a weak additional plasmon mode begins to split off from the primary plasmon mode at periods near 600 nm (Fig. 5a, left of the black X). Our coupled oscillator model assumes a fixed number of resonators. Hence the emergence of this weak fourth mode is not effectively captured in our model. Based on the electromagnetic simulation and experimental spectra (diamond symbol in Fig. 5a, b), this cut-off of the middle branch, where the absorption reaches almost zero, suggests that the strong mode coupling results in an interference condition that prevents any electromagnetic field interaction with the lossy dielectric part. This is evident from the field line profile (Fig. 5d). As compared with the UPB and LPB of absorption resonances where all the electric-field lines are recirculating or focused in the WS_2_ resonators (Fig. 5d triangle and cross), in the cut-off middle branch region (absorption dip), most electric-field lines are pushed out of or take the shortest part of interaction through the resonator, resulting in an electromagnetically ‘invisible’ or transparent resonator, qualitatively analogous to atomic systems^37,38^ (Fig. 5d diamond). Such electromagnetic ‘invisibility’ has not been observed in any lossy photonic system let alone an excitonic, external cavity-less system. Lossy grating structures have been heavily investigated for Si, Ge, and lossy metals based structures^39–43^ for the purposes of light trapping and structural coloring. In all cases, electromagnetic wave simulations suggest field lines concentrating (focused) inside the lossy dielectric part^44^ in contrast to our excitonic grating system where the field lines are pushed out of the lossy resonator part (Fig. 5d).

## Discussion

The evolution of the strong coupling with thickness (Supplementary Note 9) and resonator width (Fig. 5e) shows that there exists a critical range of resonator widths (50–330 nm) and a critical thickness (15 nm) under which this three-oscillator coupling resulting in cut-off of the MPB is supported. The field profile of the high-Q plasmon mode (Fig. 5e inset) shows significant leakage out of the TMDC, indicating sensitivity to the dielectric environment, with potential applications in chemical and biomolecular sensing^45^ as well as coupling/tuning quantum emitters in other TMDCs or h-BN^46–48^. Because the grating mode’s field enhancement is localized at the top-corners of the resonator, the grating width is particularly critical for strong-coupling. For *p* = 600 nm and widths larger than 330 nm, since the location of the two modes are too separated, the grating mode lacks the momentum to strongly interact with the exciton, which can explain the fact that no more strong interaction occurs at the point where the grate width is larger than 330 nm. Additionally, the strength of the polariton can be reduced when one dimension of the grating is limited. However, the LEP oscillator strength is much more robust compared to the pure excitons at widths lower than 300 nm (Fig. 5e, f). In comparison to the strongly coupled case (*t* < 15 nm), the pure exciton absorption almost vanishes in the uncoupled regime (*t* > 15 nm) (Fig. 5f). For these thicknesses of WS_2_, the exciton does not couple to the cavity mode to form hybrid polaritons. The exciton mode at *λ* ~ 600 nm almost vanishes in strength before it can even interact with the grating mode due to its instability in the discontinuous WS_2_ structure (Fig. 5f). Modes B and C’ correspond to the 2nd and 3rd order plasmon modes while mode D corresponds to the localized plasmonic mode. This indicates that the exciton-polaritons (UEP and LEP modes) or hybrid particles have a much greater robustness and stability to strongly couple to a second photon as opposed to pure excitons. We further stress that in our experiments and observation we observe coupling between purely optical as well as optical and matter resonances. While the latter case is clear and well-studied in both atomic, molecular, and condensed matter systems, the former case has also been a subject of active study^49,50^. For our unpatterned case, we observe coupling between optical and matter resonances as discussed above. However, as we modify the geometry of the system (patterned case), the geometric dispersion plays an important role causing optical fields to mix and couple further which results in the suppression of the mode vs non-interaction as seen in Fig. 5a vs 5f, respectively.

Our results highlight a previously unexplored aspect of TMDC excitons and optics, and opens a regime in exploring strong light–matter interactions where the TMDC serves as a host for both excitonic and cavity modes, without the need for an external cavity. Prior studies have focused on either monolayers or very thick (>100 nm) TMDCs^15^. Our results show that in this intermediate thickness regime, there is possibility of combining multiple classes of light–matter interactions (plasmons, excitons and cavity photons) resulting in strong-coupling phenomena involving two photons or three oscillators resulting in observation of optical invisibility conditions^51^. Further, our results showing a large effective separation energy (~410 meV) of the modes at room temperatures can help serve as a platform for exploring a rich diversity of polaritonic phenomena. While multilayer TMDCs are not emissive (see Supplementary Note 4), they can serve as underlying couplers for quantum point defects or other emitting gain media on top, potentially paving a way to exploring interaction between multiple quantum emitters, polariton condensation in molecular or quantum dot assemblies^52,53^ as well as low-threshold lasers at room temperatures^48,52,54,55^.

## Methods

### Sample fabrication

Measurements were performed on a template stripped Au substrate^16^. To ensure a flat surface of the gold back reflector rather than the rough surface of the evaporated gold, an epoxy-based peeling procedure is applied to the 100 nm-thick Au film evaporated on a clean polished Si wafer (parent wafer) using an e-beam evaporator (Kurt J. Lesker PVD 75). A piece of silicon wafer (transfer wafer) is glued to the noble metal film using a thin layer of thermal epoxy (Epo-Tek 375, Epoxy Technology) and then peeled upwards after the epoxy layer achieves its final hardness, resulting in stripping of the gold film. WS_2_ was mechanically exfoliated from bulk crystal (HQ-graphene) using Scotch Tape and transferred onto the Au substrate. Flakes with different thicknesses (6–30 nm) were transferred onto the substrate and identified using an optical microscope under reflected white light illumination.

Patterning of the WS_2_ flakes was achieved by a combination of electron-beam lithography (Elionix ELS-7500EX), exposure of poly methyl metha acrylate (PMMA A4) and dry etching process. Dry-etching was performed using a combination of XeF_2_ and Ar for 210 s, which is suitable for all the samples with varying thickness from 6 nm to 20 nm to be fully etched. Since XeF_2_ is highly selective, the etching process does not affect the Au substrate. Finally, the samples were cleaned in acetone to remove the 250 nm-thick PMMA resist. The grating region exhibits a clear change in color compared to the unpatterned region when viewed under an optical microscope in reflection mode under white-light illumination.

### Optical characterization

The WS_2_ reflectance spectra were obtained using a microscope with external white-light illumination (AvaLight-HAL) under normal incidence. The reflected light signals were collected from the microscope objective (Olympus SLMPLN 50X N.A. = 0.35) and analyzed using a grating spectrometer coupled to a Si focal plane array (FPA). All these instruments are integrated in the LabRAM HR Evolution Confocal Microscope. In order to avoid the signals from the system background and the gold bulk plasmon resonances, the reflectance spectra are normalized by the reflectance of a silver mirror (Thorlabs PF30-03-P01) which can be considered a spectrally uniform high reflector. While the reflectance from the Ag reference mirror is not unity, the minor (few-percent) absorption by the reference mirror will translate to a similarly minor uncertainty in absolute reflectance calibrations. We assume minimum scattering of the incident light due to normal incidence illumination, small sample roughness and very thin features compared to wavelengths. The separated TM and TE polarization signals are obtained using a linear-polarizer (Edmund Linear Glass Polarizing Filter #43-783) for the incident white light. The incident white-light spot size is ~4 μm in diameter (shown in Supplementary Fig. 2a).

### Simulations

Numerical simulations of the electromagnetic response of the WS_2_ gratings were achieved with the Lumerical FDTD and COMSOL Multiphysics solvers. 1-dimensional heterostructures were solved using the transfer matrix method. The reflectance spectra and field distributions were calculated by simulating incident plane waves with TM or TE polarizations. The anisotropic permittivity of bulk WS_2_ reported in ref. ^5,15^ was adapted for our simulations.

## Supplementary information

Supplementary Information

Description of Additional Supplementary File

Supplementary Video 1

## Data Availability

The data that support the conclusions of this study are available from the corresponding author upon request.

## Code availability

The Python scripts used in this study for plotting and modeling are available from the corresponding author upon request.
