# Supplementary material for: Hybrid exciton-plasmon-polaritons in van der Waals semiconductor gratings
Source: Nat Commun. 2020 Jul 15;11:3552. doi: 10.1038/s41467-020-17313-2 (PMC7363824; doi:10.1038/s41467-020-17313-2)
Supplement: Supplementary file 1 — Supplementary Information [file 41467_2020_17313_MOESM1_ESM.pdf]

Supplementary Information

Hybrid Exciton-Plasmon-Polaritons in van der Waals Semiconductor Gratings

Zhang et al.

Supplementary Notes 1

Evolution of the exciton and cavity modes in various semiconductor materials on Au[1]

Simulated reflection spectra for different dielectric materials (WS_2, WSe_2, GaP and GaAs) on different plasmonic substrates (Au, Ag and Al) as thickness is swept are shown in Figure S3. The more lossy the plasmonic substrate is (Al>Au>Ag), the higher Fabry-Perot mode resonance absorption the dielectric-metal heterostructure has (in the case of WS_2 on Au, Au absorbance averages ~0.2 when $t < t_{cr}$ and ~0.1 when $t > t_{cr}$). In other words, a lossy metal substrate (such as Al) can generate an even stronger cavity mode absorption below the exciton gap. The presence of the high exciton resonance in TMD materials makes WS_2 and WSe_2 good candidates to demonstrate the strong coupling regime in the visible-light range, compared with normal III-V semiconductors like GaP and GaAs.

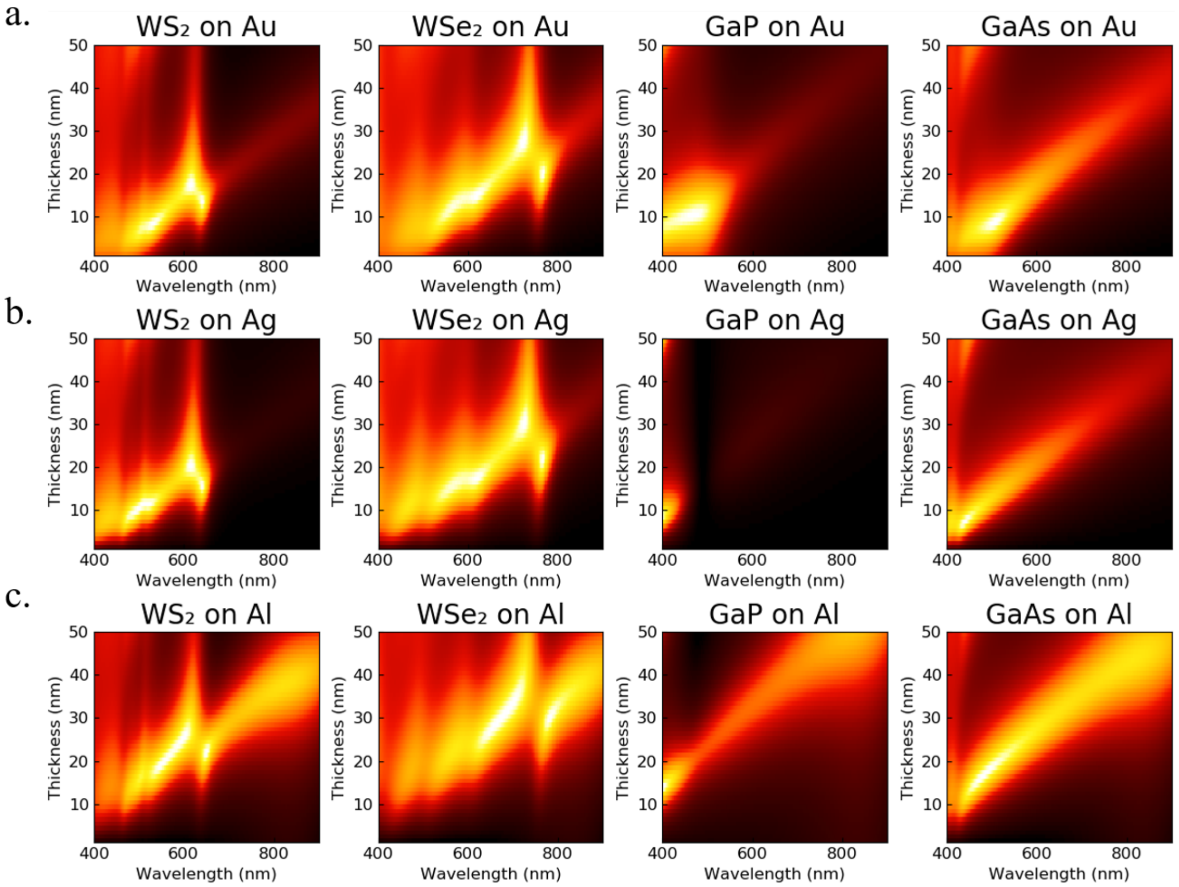

Supplementary Figure 1 Evolution of the exciton and cavity modes in various material systems Simulated absorption spectra sweep for WS_2, WSe_2, GaP, GaAs on Au (column a), Ag (column b), Al (column c).

Supplementary Notes 2

Optical images, SEM and AFM profiles of the grating structure

Extended optical images for WS_2 grating structures with various thicknesses are shown in Supplementary Figure 2 a, b, c. Unpatterned WS_2 flakes with various colors illustrate different thicknesses. The grating patterns with different width and period sizes (width varies from 200 nm to 300 nm and period varies from 500 nm to 750 nm) on the same flake show clearly the

width and period dependence of the reflectance spectral response. The AFM height profile of the specific region in Supplementary Figure 2.d is shown in Supplementary Figure 2 e. The sizes of the width (300 nm), period (600 nm) and thickness (15 nm) can be identified through the AFM image and profile. An SEM image with normal incidence is pictured in Supplementary Figure 2 f. The AFM image (Supplementary Figure 2.d) and the SEM image (Supplementary Figure 2.f) show some roughness on the edges of the grating which may explain the small deviations of the experimentally measured reflectance from simulations.

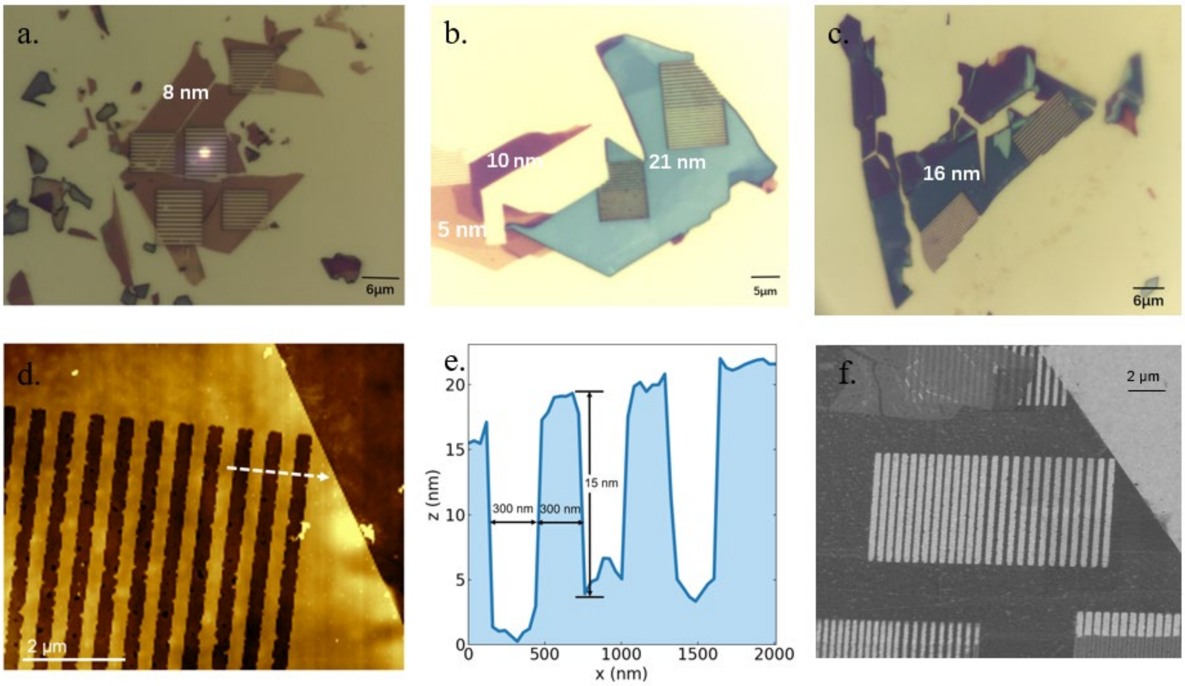

Supplementary Figure 2 Optical images (a, b, c), AFM profiles (d, e) and SEM (f) of the grating structure

Supplementary Notes 3

Control reflectance and photoluminescence measurements of WS_2 on Au

The control absorption spectra are shown below in Supplementary Figure 3 a. The bare multilayers with thickness of 5 nm, 14 nm and 21 nm are shown for comparison. The exciton-polariton splitting can be clearly seen for the 14 nm thick flake. Figure S3 b shows the PL spectrum of the multilayer WS_2 with no PL signals since multilayer TMDCs are in-direct band gap materials.

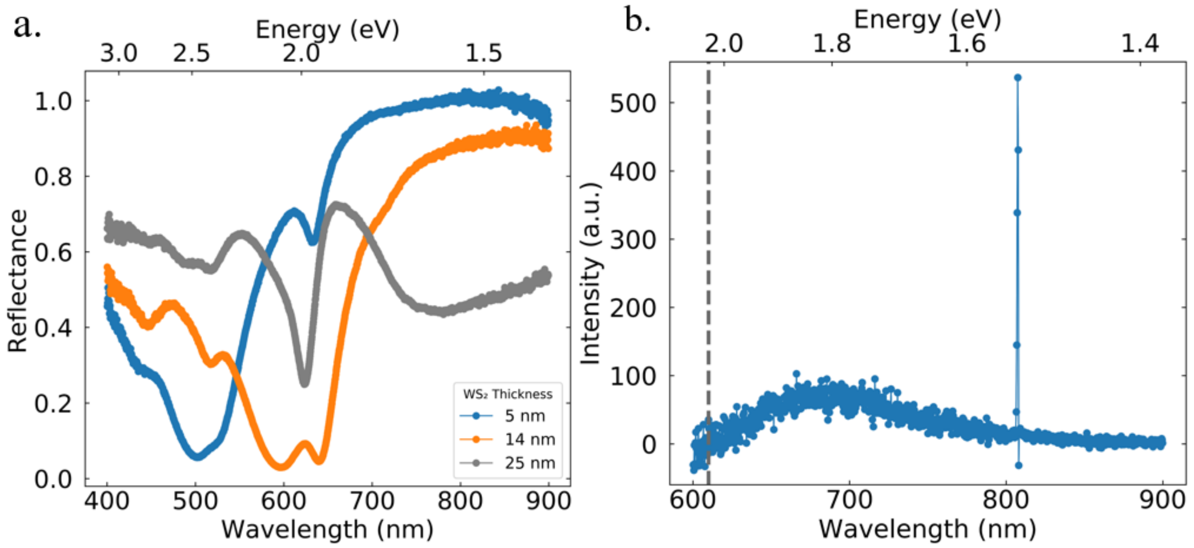

Supplementary Figure 3 Absorption (a) and photoluminescence emission (b) spectra for unpatterned few-layers of WS_2 on Au. For the absorption spectra in a, the bare WS_2 multilayers with varying thickness of 5 nm, 14 nm and 21 nm are shown for comparison. b shows the PL spectrum of a multilayer WS_2 (~20 nm) with no measurable PL signals since there is no direct bandgap in multilayer samples. The sharp line at 808 nm is the multiple of 404 nm pump laser excitation. PL spectra for all our WS2 samples with varying thicknesses look identical to the spectrum shown in b.

Supplementary Notes 4

Two-oscillator model fitting of the exciton polariton detuned by thickness[2-4]

The thickness-dependent reflectance spectra clearly show a typical anti-crossing behavior and the formation of the cavity polaritons (Figure 3.a). By fitting the simulation spectra into the calculated coupled two-oscillator model (Supplementary Figure 4), the evolution of the strong coupling can be obtained by detuning the thickness. The dashed lines in the simulation spectra represent the calculated two-oscillator fitting model which can be represented as: $\begin{pmatrix} E_C & g_{EC} \\ g_{EC} & E_E \end{pmatrix}\begin{pmatrix}\alpha \\ \beta\end{pmatrix} = E\begin{pmatrix}\alpha \\ \beta\end{pmatrix}$, where g_{EC} represents the coupling strength between the exciton mode and the cavity mode. The exciton mode and cavity mode evolve into the exciton-like polariton and photon-like polariton respectively by strong coupling. At zero detuning, the critical thickness is 15 nm and the Rabi splitting is evaluated as $\hbar\Omega$~170 meV (marked as the arrow in the plot below).

Taking thickness as t=15nm as an example, based on the simulation and calculation results in S4, the linewidth of bare exciton mode and cavity mode are γ = 124 meV and κ = 306 meV respectively. The exciton polariton splitting (2g) is around 170 meV, as the calculation indicated, 2g<(γ+κ)/2, falling into the weak coupling regime[5].

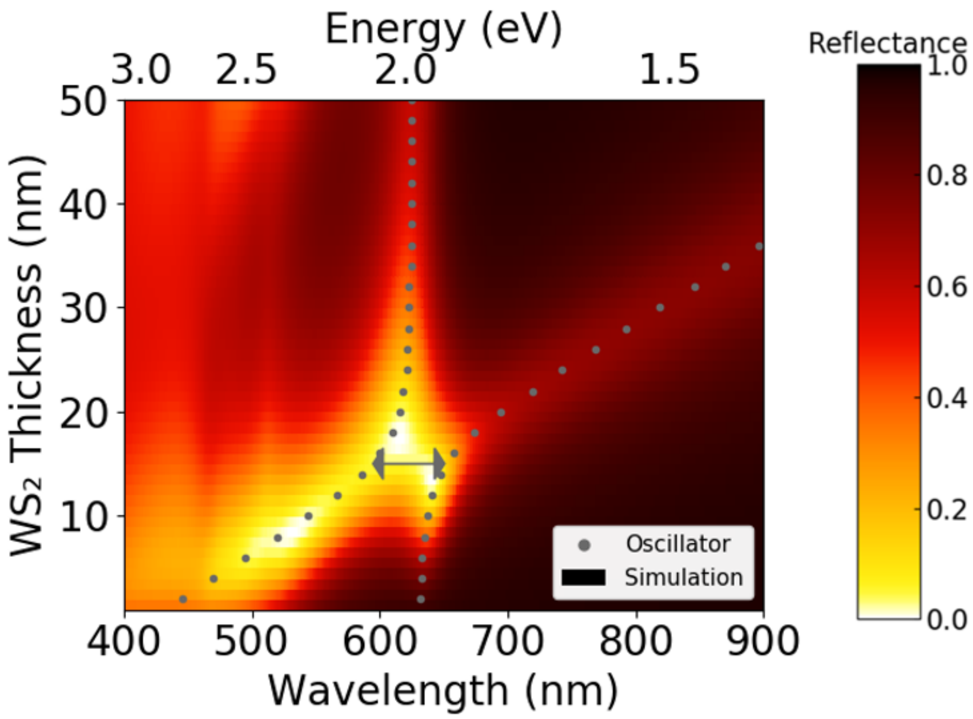

Supplementary Figure 4 Two-oscillator model fitting of the exciton polariton detuned by thickness

Supplementary Notes 5

Evolution of exciton polariton coupling in unpatterned case as function of WS_2 thickness.

The dashed line in the Supplementary Figure 5 below represents the bare exciton mode. A clear trend of mode coupling/Rabi-splitting like features is observed repetitively in the thickness dimensions suggesting that the WS_2 performs the function of an optical cavity with resonant modes at periodic thicknesses. The experiments are limited to thicknesses between 8-25 nm that represent the lowest/first order cavity mode while the higher order modes can be seen for thickness > 50 nm in the figure above.

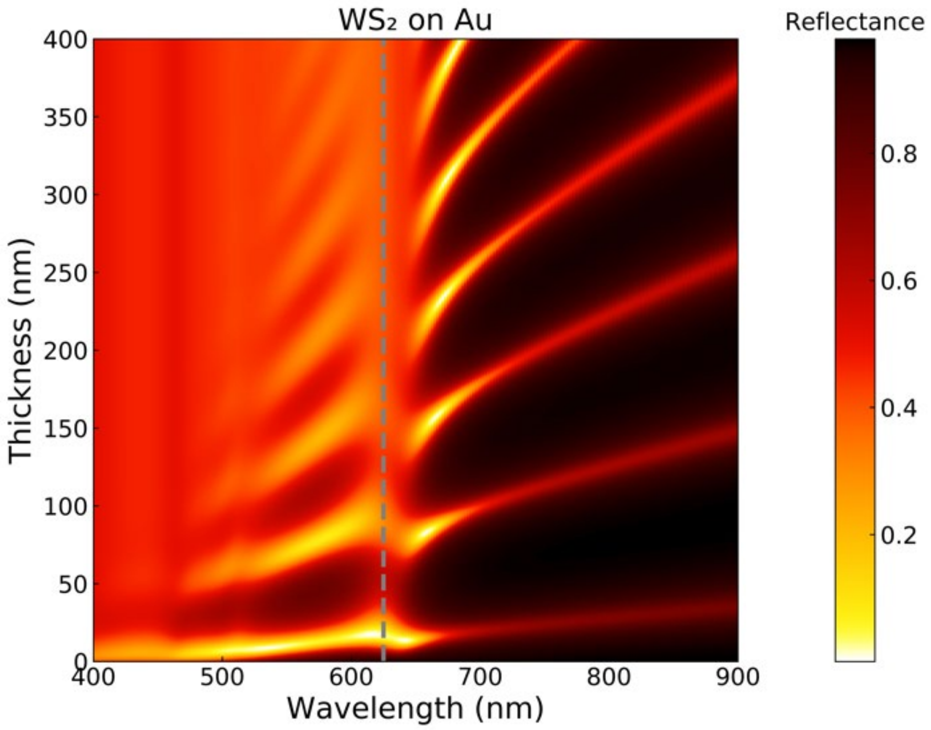

Supplementary Figure 5 Reflectance spectra of WS_2 on Au for varying WS_2 thickness.

Supplementary Notes 6

Mode evolution in TE polarization

Simulated reflection spectra in TE polarization as thickness is varied is shown in Supplementary Figure 6. a. The experimental reflectance spectra with various thicknesses (10 nm, 14 nm, 20 nm) in TE polarization (Supplementary Figure 6. b) match well with the simulation both qualitatively and quantitatively by the coded circular markers. Supplementary Figure 5.c shows field profiles of the various plasmonic modes observed in TE polarization. Here, the E_Y field profile in TE polarization corresponds to H_Y in TM polarization while H_z in TE polarization corresponds to E_Z in TM polarization.

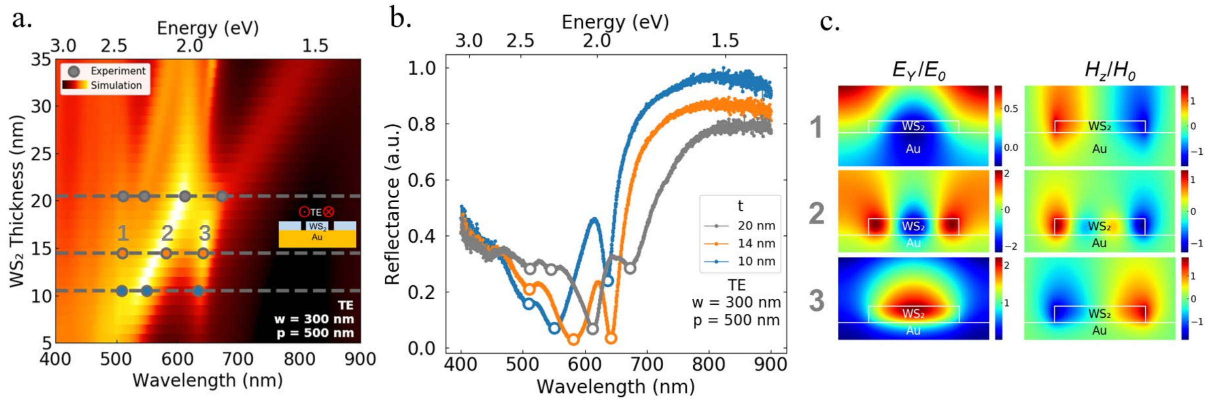

Supplementary Figure 6 Mode evolution in TE polarization. (**a**) Simulated reflectance spectra in TE polarization. (**b**) Experimental reflectance spectra. (**c**) Field profiles of the plasmonic modes.

Supplementary Notes 7

Evolution of hybrid plasmon modes based on width dependence

The animation from Supplementary Figure 7. a to i shows the evolution of the plasmon mode peak as the width of WS_2 keeps increasing. The SPR branch redshifts and higher-order SPR modes keep emerging as the width is increased (see Supplementary video/Animation 1).

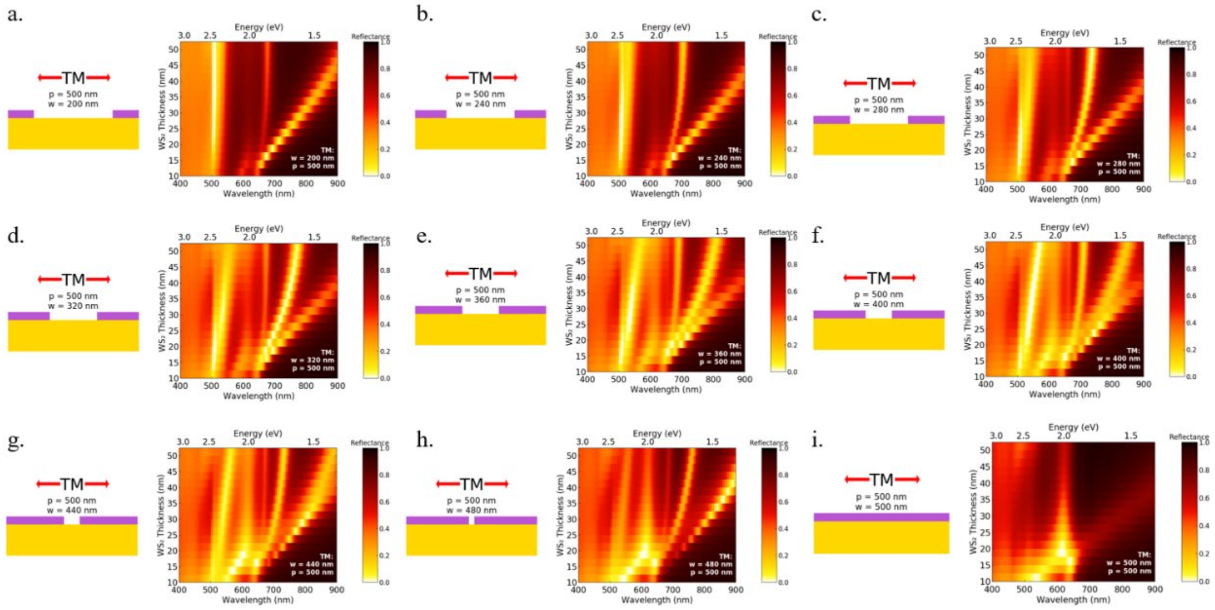

Supplementary Figure 7 (**a**) to (**i**) Evolution of SPR modes based on width dependence

Supplementary Notes 8

Three-oscillator model calculation and agreement with simulations and experiments[6,7]

We model our system as a system of three coupled oscillators as shown in Supplementary Figure 8 below. Oscillator 1 represents the grating mode, while 2 and 3 represent the two exciton polaritons (upper exciton-polariton (UEP) and the lower exciton-polariton (LEP) in figure 3.a). Assuming a sinusoidal driving force, a damping force, and a Hooke's law force, we write down Newton's law.

$$\ddot{\vec{\mathbf{x}}} + 2\overleftrightarrow{\mathbf{G}}\dot{\vec{\mathbf{x}}} + \overleftrightarrow{\mathbf{K}}\vec{\mathbf{x}} = \vec{\mathbf{F}}e^{i\omega t} \tag{1}$$

The diagonal terms of coupling matrix $\overleftrightarrow{\mathbf{K}}$ represent the eigen-resonances of the participating modes. The off-diagonal terms represent strengths of inter-mode coupling. In our case modes 1 and 3 are uncoupled, as the polaritonic coupling is dominant. Thus, we obtain:

$$\overleftrightarrow{\mathbf{K}} = \begin{pmatrix} k_1 & -k_{12} & 0 \\ -k_{12} & k_2 & -k_{23} \\ 0 & -k_{23} & k_3 \end{pmatrix} \tag{2}$$

In the uncoupled limit (i.e., with no excitons in the system), the grating mode resonance varies linearly with period. Hence k_1 take the form of an inverse square function of period by dimensional analysis (a, b, and c are fitting constants).

$$k_1(p) = \frac{a}{(bp+c)^2} \tag{3}$$

The damping matrix $\overleftrightarrow{\mathbf{G}}$ can is a diagonal matrix corresponding to decay of the excited modes.

$$\overleftrightarrow{\mathbf{G}} = \begin{pmatrix} g_1 & 0 & 0 \\ 0 & g_2 & 0 \\ 0 & 0 & g_3 \end{pmatrix} \tag{4}$$

To get a sense of absorption for the optical system, we solve the differential equation for $\vec{\mathbf{x}}(t)$ and calculate the time average energy as a function of $\overleftrightarrow{\mathbf{G}}$, $\overleftrightarrow{\mathbf{K}}$, $\vec{\mathbf{F}}$, and driven frequency ω.

Remarkably, these coupled oscillator spectra shown in Figure 5c of the manuscript are able to replicate the cut-off of exciton absorption we observe in experiment and electromagnetic simulation (Figure 5a of the manuscript). It is worth noting that in the three-oscillator model, only when we assume that the driving force (excitation) is on the second oscillator (which is the Upper Exciton Polariton (UEP) branch, F_2) can we get the resulting optical response that the inherently strong UEP absorption resonance is completely suppressed. As shown in Supplementary Figure 8, we find that the cut-off condition occurs only when oscillator 2, coupled to both the other oscillators (1 and 3), is excited. To generate the dispersion spectra in Figures 5.a and 5.c, only F_2 was nonzero.

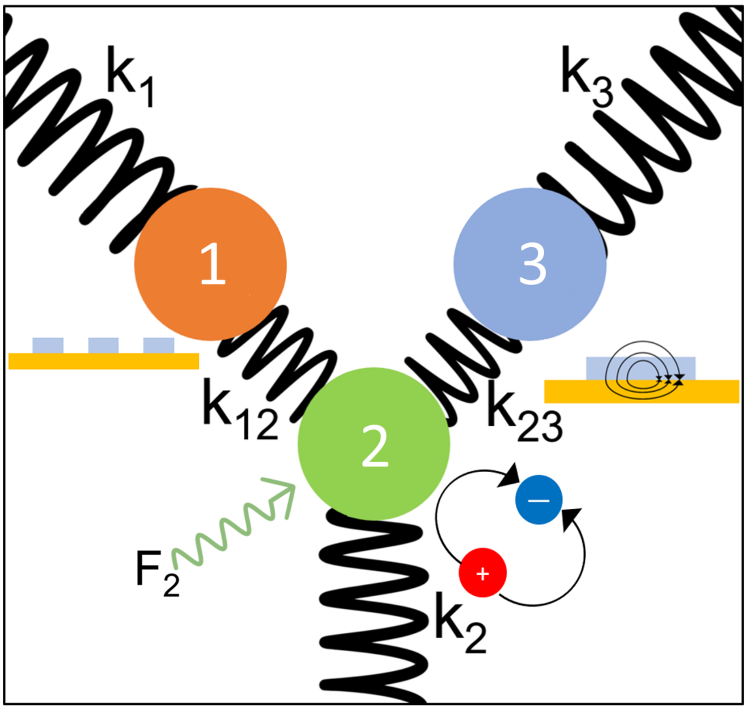

Supplementary Figure 8 Schematic description of the observed modes (oscillators) as mechanical equivalent resonators coupled with springs.

Supplementary Notes 9

Evolution of coupling as a function of thickness

The evolution of the coupling strength as a function of thickness is shown in the experimental reflectance spectra in Supplementary Figure 9. a. At the zero detuning point where the width equals 300 nm and the period equals 600 nm, the strong coupling evolves with thickness. Reflectance spectra for gratings with fixed w = 300 nm and p = 600 nm and widths of 7 nm and 11 nm are plotted. Superimposing the experimental peaks on the simulation results (Supplementary Figure 9. b), it is shown that both the d-grating peak (marked as triangles) and the plasmon peak (marked as crosses) redshift as the thickness increases.

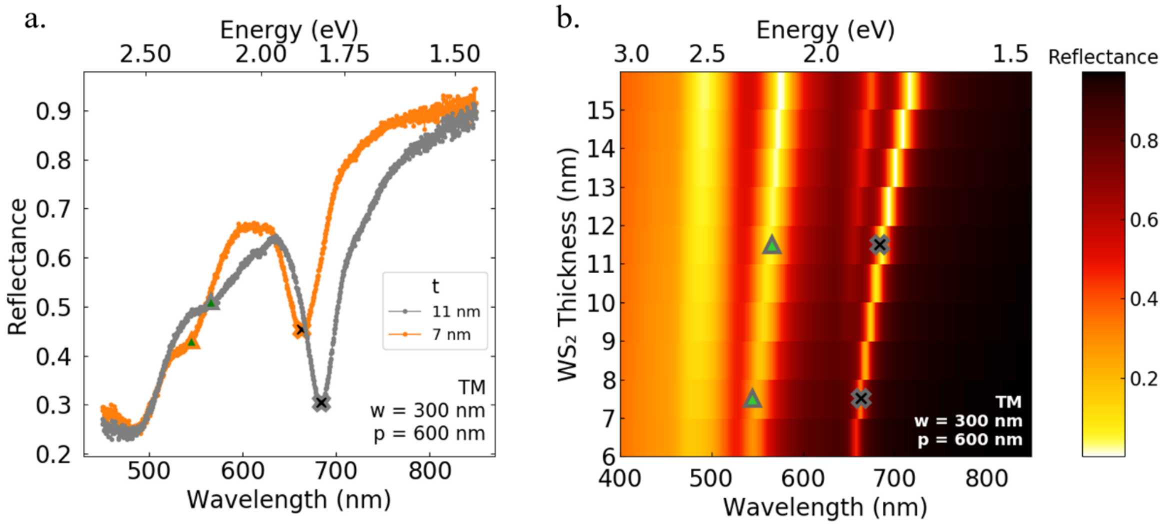

Supplementary Figure 9 Evolution of coupling as a function of thickness (a) Experimental reflectance spectra (b) Simulation reflectance spectra sweep

References:

1 Jariwala, D. *et al.* Near-unity absorption in van der Waals semiconductors for ultrathin optoelectronics. *Nano letters* 16, 5482-5487 (2016).

2 Wang, S. *et al.* Coherent coupling of WS2 monolayers with metallic photonic nanostructures at room temperature. *Nano letters* 16, 4368-4374 (2016).

3 Zhang, L., Gogna, R., Burg, W., Tutuc, E. & Deng, H. Photonic-crystal exciton-polaritons in monolayer semiconductors. *Nature communications* 9, 713 (2018).

4 Wang, Q. *et al.* Direct observation of strong light-exciton coupling in thin WS 2 flakes. *Optics express* 24, 7151-7157 (2016).

5 Khitrova, G., Gibbs, H., Kira, M., Koch, S. W. & Scherer, A. Vacuum Rabi splitting in semiconductors. *Nature Physics* 2, 81-90 (2006).

6 Qian, C. *et al.* Two-photon Rabi splitting in a coupled system of a nanocavity and exciton complexes. *Physical review letters* 120, 213901 (2018).

7 Jiang, P. *et al.* Tunable strong exciton–plasmon–exciton coupling in WS 2–J-aggregates–plasmonic nanocavity. *Optics Express* 27, 16613-16623 (2019).
