# Supplementary material for: Hybrid exciton-plasmon-polaritons in van der Waals semiconductor gratings
Source: Nat Commun. 2020 Jul 15;11:3552. doi: 10.1038/s41467-020-17313-2 (PMC7363824; doi:10.1038/s41467-020-17313-2)
Supplement: Supplementary file 2 — Description of Additional Supplementary File [file 41467_2020_17313_MOESM2_ESM.pdf]

Description of Additional Supplementary File

Supplementary Video 1

Description: Evolution of hybrid modes dispersion with WS2 resonator width (200-500 nm) at a fixed period of 500 nm and thickness ranging from 10-50 nm. Higher orders of surface plasmon resonances emerge as the width increases.
