# Supplementary figures and images for: Hybrid exciton-plasmon-polaritons in van der Waals semiconductor gratings
Source: Nat Commun. 2020 Jul 15;11:3552. doi: 10.1038/s41467-020-17313-2 (PMC7363824; doi:10.1038/s41467-020-17313-2)

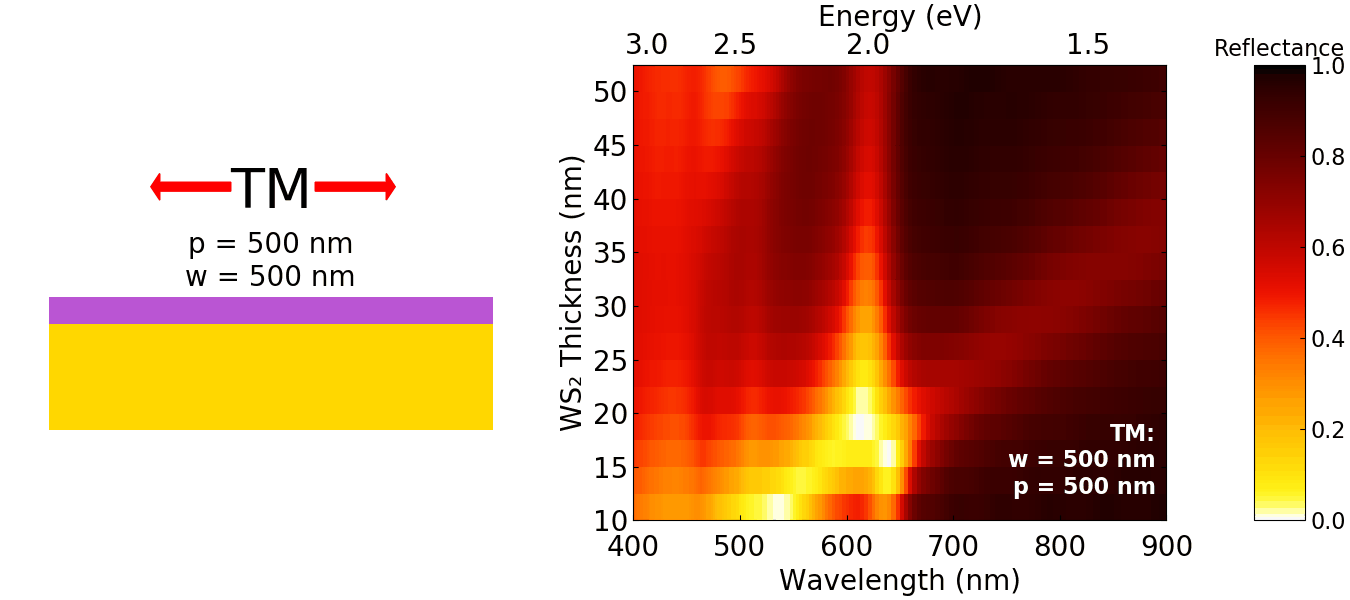

Supplement: Supplementary file 3 — Supplementary Video 1 [file 41467_2020_17313_MOESM3_ESM.gif]
